# Implementation and validation of 2-D-array based tests in routine linac quality assurance

**DOI:** 10.1016/j.zemedi.2024.01.006

**Published:** 2024-02-16

**Authors:** Jiří Valenta, Manfred Schmidt, Christoph Bert

**Affiliations:** Department of Radiation Oncology, Universitätsklinikum Erlangen, Friedrich-Alexander-Universität Erlangen-Nürnberg (FAU), Universitätsstraße 27, 91054 Erlangen, Germany; Comprehensive Cancer Center Erlangen-EMN (CCC ER-EMN), Östliche Stadtmauerstraße 30, 91054 Erlangen, Germany

**Keywords:** Linear accelerator quality assurance, 2-D array, Test sensitivity, Test specificity

## Abstract

**Purpose:**

In medical linac quality assurance (QA), to replace film dosimetry with low-resolution 2-D ionization chamber array measurements, to validate the procedures, and to perform a comprehensive sensitivity analysis.

**Methods:**

A 2-D ionization chamber array with a spatial resolution of 7.62 mm was deployed to perform the following tests: Junction tests, MLC transmission test, beam profile constancy vs. gantry angle test, beam profile constancy vs. low dose delivery test, and beam energy constancy vs. low dose delivery test. Test validation and sensitivity analyses based on short- and long-term statistics of the test results were performed.

**Results:**

All selected mechanical and dosimetry tests could be successfully performed with a 2-D array. Considering the tolerance limits recommended by the AAPM Task Group 142 report (2009), sensitivities of 99.0% or better and specificities ranging from 99.5% to 99.9% could be achieved in all tests when the proper metrics were chosen.

**Conclusions:**

The results showed that a low-resolution 2-D ionization chamber array could replace film dosimetry without having to sacrifice high test sensitivity. Its implementation in the routine clinical linac QA program may involve considerable QA time savings.

## 1 Introduction

A comprehensive linear accelerator (linac) quality assurance (QA) program is a crucial component of safe radiation therapy. Modern linacs offer built-in QA procedures with the aim to assure the specified machine performance [1], [2] allowing for significant time saving along with high levels of standardization and reproducibility. These tests use hardware and software parts provided by linac manufacturers, some of which being inseparable components of the machine under test, e.g., the linac monitor chambers or the Electronic Portal Imaging Devices (EPID). Thus, their performance relies on internal settings and/or calibration, often without influence of the local clinical staff.

This may be an attractive, comfortable, and time saving approach. Nevertheless, independent quality assurance through third-party equipment, performed and interpreted by local medical physics experts should complement the build-in QA procedures. In some countries, such independent QA procedures might be obligatory and/or required by the authorities.

Various 2-D ionization chamber arrays have been employed for machine QA to evaluate multiple linac parameters. Ritter [3] developed a 14-field test plan for the MaxtriXX Evolution (IBA Dosimetry, Schwarzenbruck, Germany) to test 13 linac parameters, e.g., the output constancy, the output linearity, or the 2-D dose distribution. This set of tests significantly exceeded the monthly QA scope as recommended by AAPM TG-142 report [4] but omitted some other tests where deployment of an array would be conceivable. As another approach towards the use of 2-D arrays, Skinner [5] deployed the IC profiler (Sun Nuclear, Melbourne, FL, USA) and automation scripts to measure output, flatness, symmetry, jaw positions, gated dose constancy, energy constancy, collimator walkout, crosshair centering, and dosimetric leaf gap constancy. Kantz·[6] compared the performance of three arrays from different manufacturers in a large number of QA tests, focusing on IMRT and VMAT delivery QA. Other authors used 2-D arrays to measure beam profile parameters and have demonstrated that they are suitable metrics for photon beam energy constancy [7], [8]. The suitability of a 2-D ionization chamber array (MaxtriXX Evolution) for detection of small leaf positioning errors has been demonstrated in studies dealing with patient specific plan verification, e.g., Shang [9] or Visser [10].

In this work, we focus on four different types of QA tests, i.e., junction, MLC transmission, beam profile constancy, and beam energy constancy, implement them in the clinical routine, and perform statistical evaluation of their performance. The test procedures were originally developed for a C-arm linac without any built-in QA procedures. They can also complement the QA program of a linac with built-in QA procedures. And, they can be transferred to another C-arm or an O-ring linac, with extensions or reductions of the test scope given by the particular beam limiting device (BLD) characteristics. The primary purpose of this work is to replace film dosimetry in linac QA with 2-D-array based tests. At the same time, we aim to save QA time by defining the procedures in a way that allows for as many acquisitions as possible with one single setup, even for different tests. In addition, whenever possible, a single measurement is used to evaluate more than one parameter. Testing, validation, and sensitivity analysis of the novel QA procedures are an essential part of this work.

## 2 Materials and methods

### 2.1 Hardware and software

The MaxtriXX Evolution is a 2-D array of 1020 parallel-plate ionization chambers arranged in a 32 × 32 grid, except for the four positions in the corners. The ionization chamber pitch is 7.62 mm and the diameter of each chamber is 4.5 mm [11]. The device covers an area of 24.4 × 24.4 cm^2^, which is also the maximum (nominal) size of the field that can be irradiated with the device placed at an SDD (source-to-detector distance) of 100 cm. The chambers are vented and operate at the nominal bias voltage of 500 V. Each chamber’s sensitive volume is 0.08 cm^3^, the height of the sensitive volume is 5.0 mm. The reference point is located 3.5 mm below the device surface and is indicated by engravings on the housing walls. The housing material is 3.0 mm ABS Tecaran (density: 1.06 g/cm^3^) and the water equivalent absorber thickness is 3.2 mm [11].

**Figure 1:**
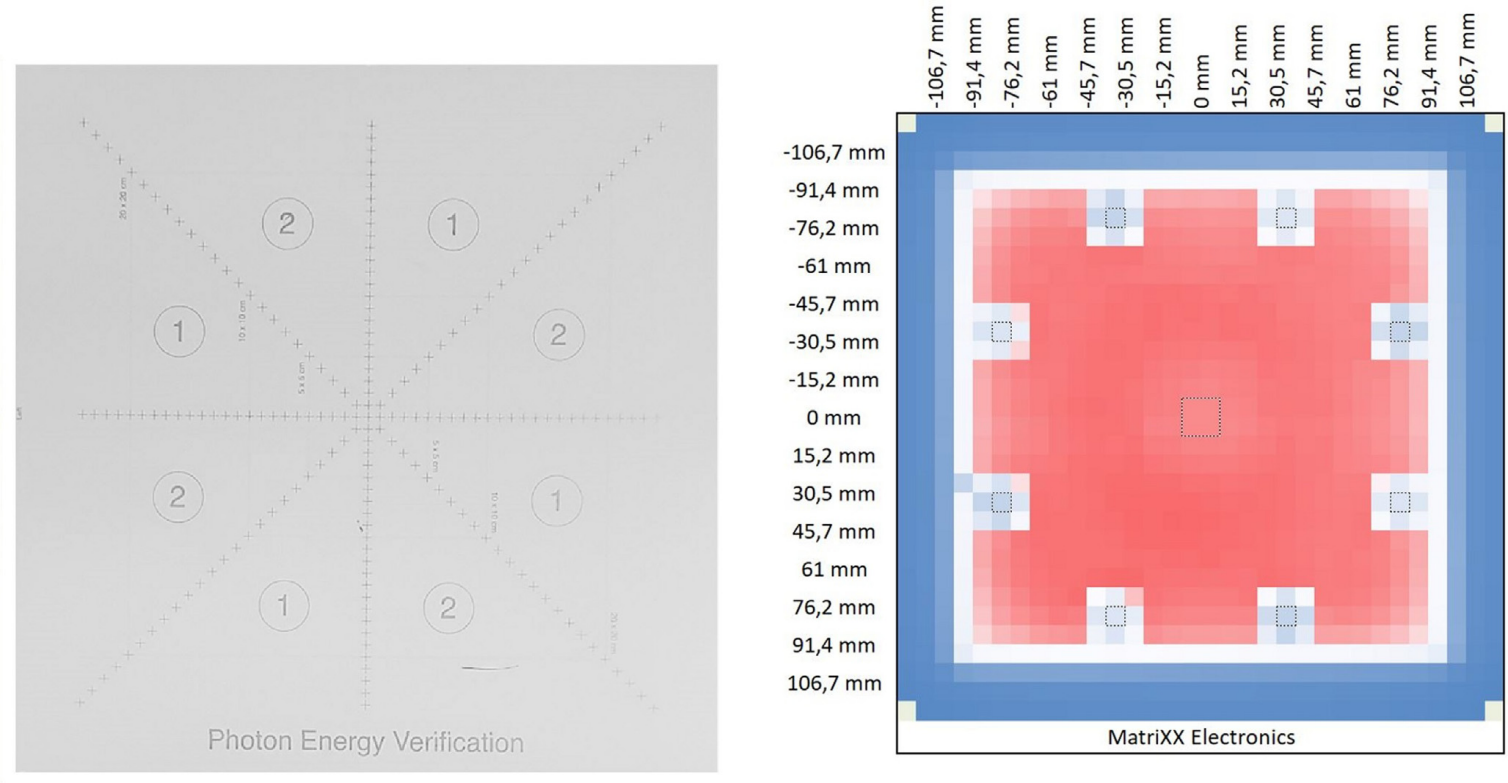
2-D array. Left: Detail of the Photon Energy Verification Plate: areas ① and ② indicate the positions of the cylindrical absorbers. Right: An example of a 20 × 20 cm^2^ open field measured with the MatriXX, as imported and graphically represented in MS Excel. Lower-dose areas in blue – outside the radiation field and below the cylindrical absorbers ① and ②.

As backscatter material, slabs of water equivalent polystyrene (RW3, density: 1.045 g/cm^3^) were placed under the array. As build-up material, slabs of RW3 or the Photon Energy Verification Plate (IBA Dosimetry GmbH, 5 cm thickness, RW3 – see Fig. 1) were placed on top of the array. The Photon Energy Verification Plate contains 8 cylindrical absorbers (diameter approximately 20 mm) symmetrically positioned off-axis, approximately 8.5 cm from the center of the plate, between the diagonals and the orthogonal midlines [12]. No detailed manufacturer’s description of the absorber properties was available. The authors assumed, based on CT scans of the plate, that areas ① and ② contain higher density absorber material of different thicknesses.

The data were acquired with myQA FastTrack. The myQA FastTrack is a stand-alone application module in the myQA software (IBA Dosimetry GmbH, Schwarzenbruck, Germany; version 2020_001), a part of the Machine QA Workspace. Background compensation with a 20 s sampling time was applied. All measurements were performed in Single shot / user start-stop mode: The software saves a single frame for each measurement as an integral of the signal over the entire acquisition time. The myQA FastTrack was only used for raw data acquisition and for data export, not for data analysis.

**Figure 2:**
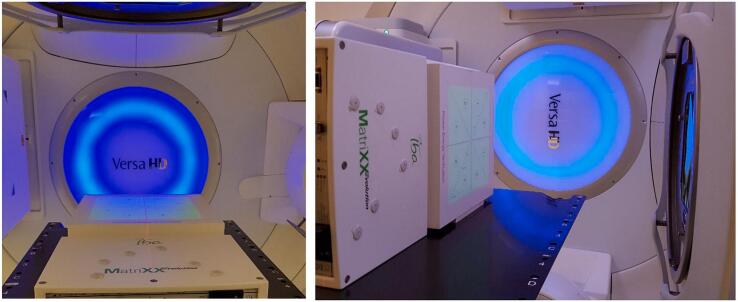
Setup. The MatriXX Evolution with the Photon Energy Verification Plate positioned upon the treatment couch for measurements at gantry angles 0° (left) and 90° (right). The greenish light field of 24 × 24 cm^2^ is visible on the plate surface.

The 2-D array was always positioned perpendicularly to the beam (see Fig. 2) at an SDD of 100 cm. 2 cm of RW3 were placed below the array as backscatter material, the build-up material was placed on top of the array. For all acquisitions at the gantry angle of 0°, it was tabletop mounted, facing the beam incidence direction, for the acquisitions at the gantry angles of 90° and 270°, it was positioned upright on its side wall upon the treatment couch. In this case, the build-up and backscatter plates were fixed with a stripe of adhesive tape.

The myQA FastTrack module allows for raw-data export of individual frames as .opg files. An .opg file is a text file containing a header and a body part, the body being a 33 × 33 matrix with the first column and the first line indicating the position in x (crossplane) or y (inplane) directions, respectively, and following 32 × 32 positions indicating the background-compensated raw signal of each of the 1020 detectors. Neither the user uniformity calibration of the device nor the air density corrections were applied. These corrections were not necessary, as the measurements were relative. The factory calibration was applied to assure uniform response of individual ionization chambers.

For the junction test validation, radiographic films (Carestream EDR2, Carestream Health, Inc., Rochester, NY, USA) were digitized with an EPSON Perfection V850 Pro scanner (Epson, Suwa, Nagano, Japan) and evaluated with FIJI, an image processing package based on ImageJ [13]. Furthermore, EPID images of respective linacs and treatment planning system (TPS) calculations (RayStation 9B, RaySearch Laboratories, Stockholm, Sweden) were compared with the array measurements.

### 2.2 Tests and setup

The QA tests were originally developed for an Elekta Versa HD linear accelerator (C-arm linac, Elekta AB, Stockholm, Sweden). They were also tested and partially implemented on further linacs: Vero (O-ring linac, BrainLAB AG, München, Germany and Mitsubishi Heavy Industries, Tokyo and Yokohama, Japan), Novalis Tx (a Varian Trilogy-series based C-arm linac, Varian Medical Systems Inc., Palo Alto, CA, USA and BrainLAB AG, München, Germany) and Halcyon (O-ring-linac, Varian Medical Systems Inc., Palo Alto, CA, USA). Due to different BLD characteristics of these linacs, e.g., missing or additional jaws or limited collimator rotation, this implementation required minor test extensions or reductions (for details, see the description of junction tests below, and the list of irradiation fields provided in the Supplementary material).

In the following QA tests, film dosimetry of photon beams was replaced by 2-D array measurements: Junction tests (complementary half-field with collimator rotation and complementary 4 quadrants), MLC transmission test, beam profile constancy (vs. gantry angle and at low dose delivery) and energy constancy at low dose delivery. This test selection was inspired by the German standards DIN 6847-5 [14] and DIN 6875-4 [15].

#### 2.2.1 Junction tests

The purpose of the junction tests is to assure the constancy of leaf and/or jaw positioning under the influence of gravity (half-field junction) or the constancy of the leaf positioning together with the dosimetric leaf gap (4-quadrant test). The dosimetric effect of the mechanical change over the junction area can be i) visualized: qualitative analysis, e.g., with a radiographic film or an EPID, and ii) measured: quantitative analysis, e.g., with a digitized radiographic film, an EPID, or a 2-D array.

The implementation deployed the setup as described above with 2 cm of RW3 as additional build-up material. Each time a reference open field of 24.0 × 24.0 cm^2^ and two complementary fields were delivered. In the 4-quadrant test, the bottom-left plus top-right and bottom-right plus top-left quadrants were combined. The 4-quadrant junction tests were implemented for all clinical beam energies at the gantry zero angle. The half-field junction tests were implemented for one (most commonly used) beam energy only, at the gantry angle of 90°. 90° and 270° collimator angles were combined to test the positioning of the Y-jaws under influence of gravity, 0° and 180° collimator angles were combined to test the MLC-leaf positioning. On linacs not allowing for collimator rotation to 180° (Novalis Tx and Halcyon), 45° and 225° collimator angles were used for both X-jaw (Novalis Tx only) and MLC-leaf positioning tests. One of the linacs (Vero) did not allow for any collimator rotation. Here, to test the MLC positioning under the influence of gravity, gantry angles of 90° and 270° were combined without repositioning the array. Thus, the beam incidence direction was from the array front side for gantry at 90° and from the back side of the array for gantry at 270°. Each time an open field of 15.0 × 15.0 cm^2^ (Vero maximum field size) at the respective gantry angle was acquired for reference.

#### 2.2.2 MLC transmission tests

MLC transmission was tested in a relative mode, i.e., the array/film reading of a fully closed MLC was compared to the data of an irradiation with an open field. Also, for these tests, 2 cm of RW3 was placed on top of the array as additional build-up. A reference open field (24 × 24 cm^2^) plus 3 different fully closed fields were delivered (overtravel with all leaves shifted to the left, overtravel with all leaves shifted to the right, and interdigitation). The MLC transmission test was implemented for all clinical beam energies at the gantry angle of 0° and the collimator angle of 0°, and for one (most commonly used) beam energy at the gantry angle of 90° with the collimator angles of 0° and 90°.

#### 2.2.3 Beam profile constancy vs. gantry angle

Constancy of the lateral dose distribution is being checked at main gantry angles (0°, 90° and 270°). Open fields of 24 × 24 cm^2^ were delivered in this test. The array was repositioned for each acquisition to face the beam incidence direction. The Photon Energy Verification Plate was placed on top of the array as build-up material to achieve reasonably flat profiles. The test was implemented for all clinical beam energies.

#### 2.2.4 Beam profile constancy vs. low dose delivery

The purpose of this test is to check the symmetry and flatness of the lateral dose distribution shortly after the beam is turned on. Low numbers of monitor units (5 MU and 15 MU) were delivered with open fields of 24 × 24 cm^2^. The Photon Energy Verification Plate was placed on top of the array as build-up material. The test was implemented for all clinical beam energies.

#### 2.2.5 Energy constancy vs. low dose delivery

The purpose of this test is to check the beam energy constancy shortly after the beam is turned on. The test was simultaneously performed to the aforementioned beam profile constancy test, therefore following the same setup (see Beam profile constancy vs. low dose delivery).

### 2.3 Data Import and Analysis

The .opg data – as exported from the myQA FastTrack module – were imported to MS Excel (Microsoft Corporation, Redmond, Washington, U.S.) using VBA (Visual Basic for Applications) macros. The mathematical operations, namely, the summation, division, or normalization of matrices, and the data analyses were performed in MS Excel.

#### 2.3.1 Junction test

In the junction tests, the signal matrices of both complementary partial fields were summed entry wise. Each entry of the summation matrix was divided by the corresponding entry of the signal matrix of an open field. The average signal along the inplane (half-field test and 4-quadrant test) and crossplane (4-quadrant test) midlines of the resulting relative-signal matrix, was compared to the baseline. Here, the average signal in areas not affected by the junctions was taken as reference.

#### 2.3.2 MLC transmission

For the MLC transmission tests, each element of the signal matrix of the fully closed field was divided by the corresponding element of the signal matrix of the open field. As the relative signal corresponds to the transmission in a particular area of the field, local maxima were analyzed.

#### 2.3.3 Beam profile constancy vs. gantry angle

In the beam profile constancy vs. gantry angle tests, the off-axis ratio (point by point), flatness, and symmetry of individual profiles taken at gantry angles of 90° and 270° were compared with a reference profile taken at gantry angle of 0°. As the array, consisting of an even number of detectors in both directions, does not have any detectors located centrally on its midlines, the average signal of two neighboring ionization chambers located next to midline was considered as one off-axis point, and the average signal of four centrally located ionization chambers was considered as the central point. The off-axis ratio was defined along the crossplane and inplane midlines as the signal at a specified distance from the beam axis over the signal at the central point. Symmetry was defined as the maximum of signal ratios at each pair of points located at the same off-axis distance, along the crossplane or inplane midlines. Flatness was defined as the ratio of the maximum over the minimum signal along the midline (crossplane or inplane). Off-axis ratio, symmetry, and flatness were evaluated along crossplane and inplane midlines over the flattened area (approximately 80% of the nominal field size).

#### 2.3.4 Beam profile constancy vs. low dose delivery

In the beam profile constancy tests for low dose delivery, the off-axis ratio, flatness, and symmetry of individual profiles (for details see paragraph Beam profile constancy vs. gantry angle) taken with 5 MU and 15 MU were compared to the reference profile taken with 100 MU.

#### 2.3.5 Energy constancy vs. low dose delivery

In the beam energy constancy tests at low dose delivery, both orthogonal and diagonal profile flatness, off-axis ratios and signal below the cylindrical absorbers of the Photon Energy Verification Plate were analyzed. 5 MU and 15 MU acquisitions were compared to the reference measurement taken with 100 MU.

### 2.4 Test Validation and Sensitivity Analysis

All tests were validated by introducing deliberate errors. Further, in junction tests, the effect of array mispositioning on the test outcome was analyzed. The validation and sensitivity analysis were performed on the Versa HD linac. Additionally, the Vero linac was deployed to validate the MLC transmission test.

A successful test validation was indicated by the sensitivity and specificity values that could be achieved with desired tolerance levels: Sensitivity, or true positive rate, i.e., the probability of detection, was defined as the ability to detect a state exceeding the tolerance levels, in percent of true positive over truly positive results (truly positive being the sum of true positive and false negative results). The miss rate, or false negative rate, equals 1 – sensitivity. Specificity, or true negative rate, was defined as the percentage of true negative over truly negative results (truly negative being the sum of true negative and false positive results). The false positive rate, i.e., the probability of false alarm, equals 1 – specificity.

**Figure 3:**
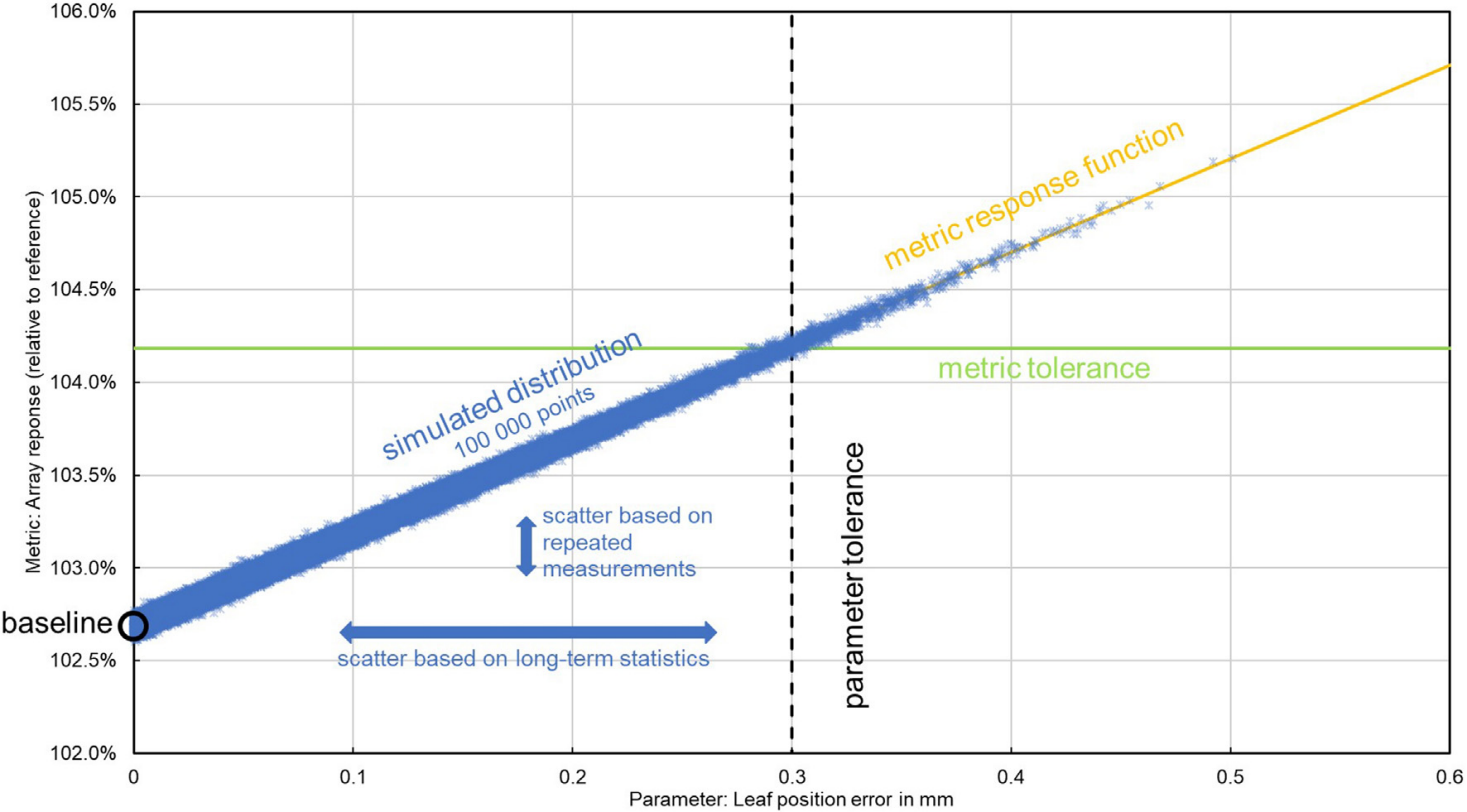
Sensitivity analysis. An example of result distribution modelling. A long- and short-term statistics-based distribution of 100 000 measurement results of leaf position error with the array response as the metric.

An example of sensitivity analysis is shown in Fig. 3. The tolerance levels were based on the AAPM Task Group 142 report [4]. For each test, the respective metric’s tolerance levels were adjusted (decreased, so that they always correspond to levels equal to or stricter than the ones given by the TG-142) to get a sensitivity of approximately 99.0%. Then, a test was considered successfully validated if the specificity was equal to or better than 99.0% as well. For the sensitivity analysis, a distribution of 100 000 test results was simulated using random numbers distributed along the metric response function as follows: Normal distribution with parameters (i.e., mean and standard deviation) taken from long- and short-term statistics was assumed: The scatter of the investigated parameter (e.g., the change in leaf-bank position, or the *TPR*_20,10_) was based on the long-term statistics of 13 measurements taken over a period of 3 years. To reflect the statistical distribution of the metric (e.g., the array response change, or the flatness), short-term statistics based on 20 measurements taken within one session was considered. The metric response function was based on measurements with deliberately introduced errors of respective parameters, for details see description of individual tests below.

#### 2.4.1 Junction test

The junction tests were analyzed by introducing deliberate leaf positioning errors creating gaps and overlaps along the inplane midline from 0.2 to 2.0 mm (in steps of 0.2 to 1.0 mm). The results of the array measurements were compared with TPS calculations and with results of other QA tools: EPID and radiographic film. The EPID images were analyzed with FIJI. The films were scanned with a flatbed scanner and evaluated with FIJI. In addition, an experienced medical physicist performed a visual (pass/fail) evaluation.

Furthermore, the effect of detector misalignment on the junction test sensitivity was investigated by shifting the array by 2, 4, 6, 8, and 10 mm in the leaf-motion direction. Same leaf positioning errors as above were introduced, creating gaps and overlaps from 0.2 to 2.0 mm.

#### 2.4.2 MLC transmission test

The MLC transmission test sensitivity was investigated by introducing holes on the inplane midline of an otherwise fully closed field. The overtravel pattern was taken for the closed part of the field. The minimum leaf separation allowed by the linac was 5.0 mm, the maximum separation introduced was 10.0 mm. To investigate the array response for holes smaller than 5.0 mm, further shielding (two lead blocks of approximately 2 cm thickness) was added, reducing the original separation to 3.0, 2.0, and (theoretically) 0.0 mm by moving the blocks closer to each other consecutively. To be more conservative, the holes were always located above an area between individual ionization chambers of the array. This was checked using the light field projection on the array surface. Additionally, MLC transmission maps as acquired with the array were compared with transmission maps acquired with a high spatial resolution detector (EPID). This was done on the Vero, the linac known for relatively inhomogeneous transmission maps compared to the other machines of the department.

#### 2.4.3 Beam profile constancy

There was no necessity to validate the MatriXX’s ability to measure beam profiles as the uniformity of individual ionization chambers is given by the MatriXX factory calibration. To analyze the sensitivity of the beam profile constancy tests, small profile changes affecting the off-axis ratio and thus both the symmetry and flatness were introduced by temporarily manipulating the linac beam steering parameters (symmetry in the crossplane direction – ‘2T Error’ changes from -3.0 to +3.0 on Elekta machines). The effect of the manipulation was analyzed. Also, several measurements without any manipulation were performed to evaluate the typical scatter under normal conditions.

#### 2.4.4 Energy constancy at low dose delivery

The energy constancy test at low-dose delivery was validated by introducing beam energy changes and comparing the response under cylindrical absorbers ① and ② of the Photon Energy Verification Plate as well as by analyzing changes to the off-axis ratio and to the beam flatness. The energy was manipulated i) by changing the gun current by approximately +/- 10% or ii) by beam hardening, i.e., placing high-Z material (lead alloy, 2 cm thickness) between the source and the detector. As the measurements with changed gun current (i) were performed on a clinical linac, saving of the manipulated settings was avoided, thus an independent determination of the beam quality using ionization chamber measurements was impossible. Therefore, no relation between the gun current change and the energy change in terms of, e.g., *TPR*_20,10_ can be reported. By using the lead alloy (ii), no modifications of the linac’s internal settings were involved, and the modified beam quality could be determined using ionization chamber measurements in the traditional setup, and reported in terms of *TPR*_20,10_.

## 3 Results

### 3.1 Test Implementation

All six mechanical and dosimetry tests could be successfully performed with a 2-D array. A total of 26 fields needed to be delivered to test one (the most commonly used) photon energy on a linac with a single-layer MLC and one pair of backup jaws. A second pair of backup jaws required 4 additional fields for the junction tests. Further fields had to be considered for a dual MLC. 11 additional fields were needed to test each additional photon beam energy.

The test sequence could be optimized in such way that one only needed to enter the treatment room four times: Thrice to reposition the array (changing between the vertical and horizontal array orientation) and once to replace the RW3 slabs with the Photon Energy Verification Plate.

### 3.2 Test Validation and Sensitivity Analysis

#### 3.2.1 Junction tests

The method was sensitive to leaf positioning errors. A leaf or jaw positioning error of 0.1 mm caused an array response change of 0.5% in the affected area. In junction tests with 180° collimator rotation, a leaf bank or jaw positioning error generated an effective gap or overlap of a doubled size, due to the addition of complementary erroneous fields. Here, a leaf or jaw positioning error of 0.1 mm (corresponding to a gap or overlap of 0.2 mm) caused an array response increase or decrease, respectively, of 1.0% in the affected area.

**Figure 4:**
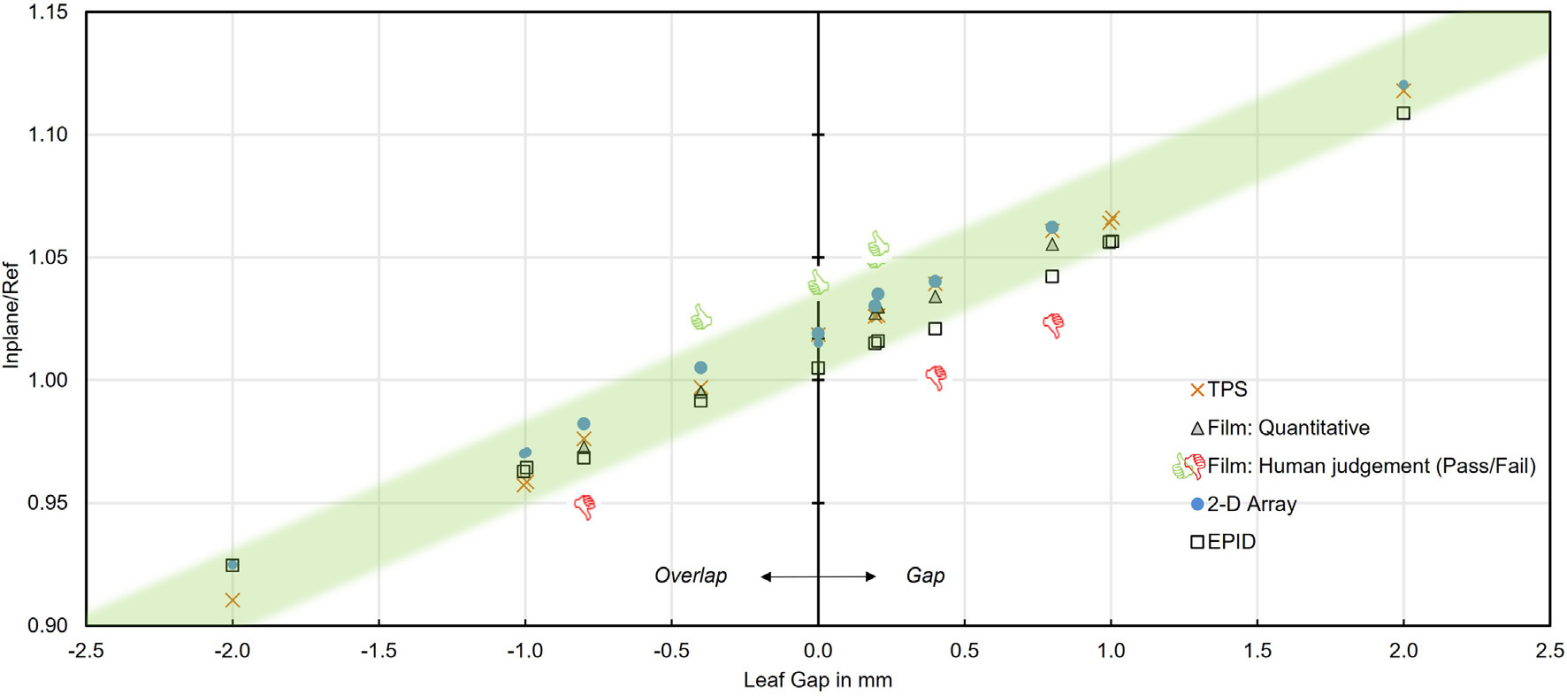
Junction test. 2-D array response over leaf gap/overlap function (circles) and its agreement with alternative QA tools (quantitative analysis of radiographic films – triangles, EPID – squares, human judgement of films – pass/fail symbol, unscaled) and with the TPS calculations (saltires). The green shaded area depicts the tolerance interval of approximately 1.5% around the TPS linear regression fit.

This dependence was linear over the entire tested range from -2.0 mm (leaf overlap) to +2.0 mm (leaf gap) as shown in Fig. 4. Similar dependence was observed for alternative QA tools, namely, EPID and digitized radiographic film, whereas the EPID response appeared marginally lower and its slope marginally smaller. All quantitative methods were in a good agreement with TPS calculations. The qualitative (pass/fail) evaluation of the radiographic film by an experienced physicist was inconsistent at one point (overlap of -0.4 mm) where the physicist’s judgement was “pass” whereas the gap (+0.4 mm) was judged as “fail”. According to actual clinical tolerance for leaf positioning of ±0.3 mm (corresponding to gaps/overlaps of 0.6 mm in adjacent fields), both +0.4 mm gap and –0.4 mm overlap would be outside the tolerance levels.

With the leaf positioning tolerance of 0.3 mm and considering the array response function as shown in Fig. 4, a reasonable array-response tolerance level would be 1.5%. In such case, the sensitivity of the test is 99.0% with a specificity better than 99.9%.

A misalignment of the array of up to 4 mm did not cause any difference in the array response function nor the sensitivity. For larger array-shifts, a decrease of the function steepness could be observed, for an 8 mm shift, the slope dropped to approximately 50% of its original value.

#### 3.2.2 MLC transmission

Transmission changes could be detected with the 2-D array. A deliberately introduced 5 mm hole within an otherwise fully closed MLC caused a change to the array response of 9% (6 MV) to 13% (15 MV). A similar response function was observed for larger gaps. For smaller gaps created by additional collimation through lead blocks, a response increase of approximately 3% per mm was observed for a 6 MV beam. Due to different collimation geometry, these results cannot be compared directly. In both cases, with a tolerance level of 0.3% from baseline, the test sensitivity and specificity were 99.1% and 99.7%, respectively.

**Figure 5:**
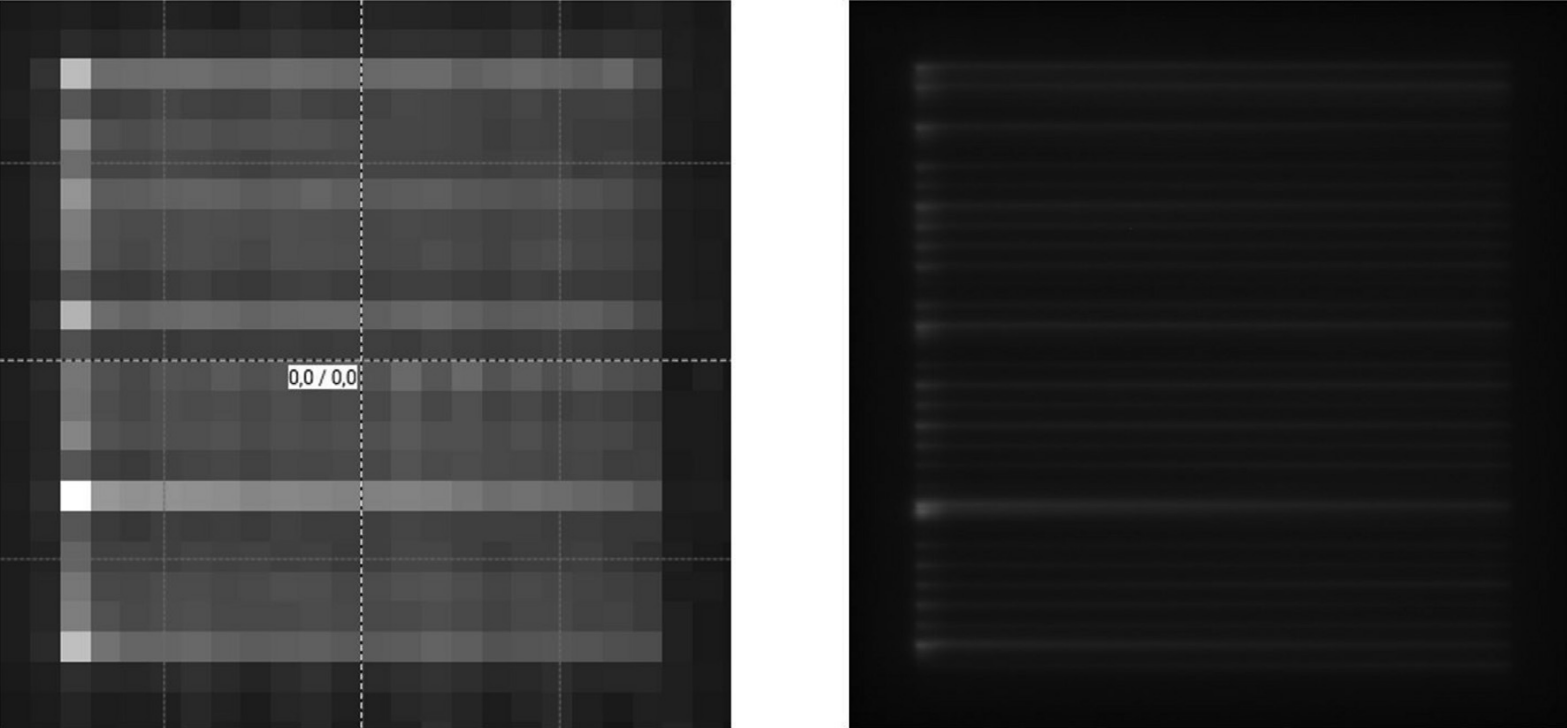
MLC transmission. Visual comparison of transmission maps acquired with a 2-D array (left) and EPID (right).

In the comparison of transmission maps acquired with the array and with the EPID (see Fig. 5), the array was overresponding in areas with high transmission on both edges of a particular leaf where both hotspots contribute to the signal of one ionization chamber of the array. It was underresponding in areas comprising a tiny hotspot surrounded by normal or low transmission. To reflect this underresponse, the array-measured transmission tolerance had to be adjusted, so that the tiny hotspots detected by an EPID as exceeding the TG-142 [4] recommended transmission tolerance of 0.5% from baseline would be detected by the array as well. Accordingly, the array transmission tolerance was set to 0.3% from baseline. Then, transmission hotspots that would appear on an EPID as exceeding the 0.5% tolerance level could be detected by the array with a 99.0% sensitivity. The specificity could still be kept at around 99.7%. Details are provided in Table 1.

**Table 1:** MLC transmission test validation for a Versa HD linac.

Beam energy	Metric	Metric tolerance	Transmission tolerance	Test sensitivity	Test specificity
6 MV	Response ratio	0.3%	0.5%	99.1%	99.7%
10 MV	Response ratio	0.3%	0.5%	99.0%	99.7%
15 MV	Response ratio	0.3%	0.5%	99.0%	99.7%

#### 3.2.3 Beam profile constancy

The typical scatter of lateral beam profile parameters observed in 20 measurements at 6 MV without manipulating the beam steering was analyzed. The standard deviations of flatness, symmetry, and maximal off-axis ratio (maxOAR) over the central 80% of the radiation field width were 0.07%, 0.14%, and 0.06%, respectively. Next, the effect of beam steering manipulation on these beam parameters was investigated. The slope of the respective response functions was 0.6%, 0.7% and 0.3% (parameter change over beam steering change (‘2T Error’) in a.u.) for flatness, symmetry and maxOAR, respectively. Flatness was the most sensitive metric of beam profile constancy due to its low scatter compared to symmetry and because of its relatively high response to beam profile changes compared to the maxOAR. Setting the tolerance level to 1.0%, sensitivity and specificity of 99.0% and 99.5%, respectively, could be achieved. For the results of other metrics see Table 2.

**Table 2:** Beam profile constancy test sensitivity analysis.

Beam energy	Metric	Metric tolerance	Test sensitivity	Test specificity
6 MV	Flatness	1.0%	99.0%	99.5%
	Symmetry	1.0%	99.0%	97.9%
	maxOAR	0.8%	99.0%	99.2%

#### 3.2.4 Beam energy constancy

The sensitivity of the beam energy constancy tests was investigated. The beam quality was modified in two ways: Changing the gun current allowed for nearly continuous variation of the mean photon energy but this kind of manipulation intrinsically affects the beam profile shape. The relation between the gun current change and the energy change in terms of, e.g., *TPR*_20,10_ could not be determined (for explanation, see the test validation description in Materials and Methods). Considering the off-axis ratio (*OAR*) change as a metric for energy change, with a tolerance level of 0.8%, a sensitivity of 99.0% with a specificity better than 99.0% could be achieved. If the diagonal flatness is taken as the metric, with a tolerance level of 1.5%, a sensitivity of 99.0% could be achieved with a specificity of 99.7%. For detailed results see Table 3.

**Table 3:** Beam energy constancy test validation: Manipulating gun current.

Beam energy	Metric	Metric tolerance	Test sensitivity	Test specificity
6 MV	*OAR* crossplane	0.8%	99.0%	99.1%
	*OAR* inplane	0.9%	99.0%	99.7%
	*OAR* absorber ①	1.0%	99.0%	99.7%
	*OAR* absorber ②	0.4%	99.0%	98.5%
	Flatness crossplane	1.0%	99.0%	99.5%
	Flatness inplane	1.0%	99.0%	98.8%
	Flatness diagonal	1.5%	99.0%	99.7%

As for the beam hardening in a 6 MV photon beam: Taking the *OAR* along the orthogonal axes as the metric for energy change, with a tolerance level of 0.8% (corresponding to a *TPR*_20,10_ change of 0.005), a sensitivity of 99.0% and a specificity better than 99.2% could be achieved. Taking the cylindrical absorbers into account instead (i.e., the response ratio under the absorbers to the central axis), sensitivity and specificity of 99.0% and 98.6%, respectively, were observed. Using the crossplane or inplane flatness change as the metric, with a tolerance level of 1.0%, a sensitivity of 99.0% with a specificity better than 98.6% could be achieved. The diagonal flatness response function was steeper than the ones of the *OAR* or the orthogonal flatness, thus, tolerance levels of 1.4% and 1.9% could be used for 6 and 10 MV, respectively, corresponding to an energy change of 0.005 in terms of *TPR*_20,10_. Then, sensitivities and specificities better than 99.0% and 99.5%, respectively, could be achieved. Details are provided in Table 4.

**Table 4:** Beam energy constancy test validation: Beam hardening.

Beam energy	Metric	Metric tolerance	*TPR*_20,10_ tolerance	Test sensitivity	Test specificity
6 MV	*OAR* crossplane	0.8%	0.005	99.0%	99.2%
	*OAR* inplane	0.8%	0.006	99.0%	99.5%
	*OAR* absorber ①	0.8%	0.007	99.0%	98.6%
	*OAR* absorber ②	0.4%	0.006	99.0%	98.8%
	Flatness crossplane	1.0%	0.005	99.0%	99.3%
	Flatness inplane	1.0%	0.007	99.0%	98.6%
	Flatness diagonal	1.4%	0.005	99.1%	99.6%
10 MV	*OAR* crossplane	1.2%	0.005	99.0%	99.5%
	*OAR* inplane	1.2%	0.006	99.0%	99.6%
	*OAR* absorber ①	1.2%	0.006	99.0%	99.7%
	*OAR* absorber ②	0.7%	0.005	99.0%	99.5%
	Flatness crossplane	1.0%	0.005	99.0%	99.3%
	Flatness inplane	0.9%	0.007	99.0%	99.4%
	Flatness diagonal	1.9%	0.005	99.0%	99.8%

## 4 Discussion

The deployment of a 2-D ionization chamber array for routine linac QA was motivated by the need of replacing film dosimetry with a novel method. It delivers results in real time and, on the contrary to radiographic films, there is no need of subsequent chemical processing. Radiographic or radiochromic films need to be scanned to enable further data processing and quantitative evaluation; in a 2-D array, on the contrary, the analog-to-digital conversion is performed in real time within the device and the delivered result are intrinsically digital. Compared to a linac built-in amorphous silicon EPID, an ionization chamber array features less dose and dose-rate dependence and a more reliable and more stable calibration in terms of absolute dose [16], [17], [18]. Diode arrays have similar advantages as ionization chamber arrays (real time and linac-manufacturer independent results) but a possibly worse time stability [17], [19]. The diode response change with accumulated dose may create further challenges as reported by, e.g., Liengsawangwong [20]. Ionization chambers feature air-density and beam quality dependences that require corrections when deployed for absolute dose measurements. The air-density response function is well known and described, e.g., by Taylor [21] or Christ [22]. The beam quality response function similar to the one of a well-established ionization chamber, CC04 (IBA Dosimetry, Schwarzenbruck, Germany) in the range of clinical linac beam energies, has been reported by Togno [17] for an array similar to the MatriXX. Ionization chamber arrays feature a good short-term stability after pre-irradiation [16], [23].

With the deployment of an ionization chamber array in the routine QA we could save the time needed for processing and digitization of films. Even more QA time can be saved by optimizing the test sequence and by using several of the acquisitions for more than one test. Before the introduction of the 2-D-array in junction tests in our department, the digitization of films was not performed routinely, and a judgement of a medical physics expert, evaluating the film with the bare eye, was determining the (pass/fail) result of the test. This qualitative method has proven to be less reliable than the quantitative evaluation of the array measurements (see Fig. 4). We have also proven that minor detector shifts have no measurable effect on the test results. This, at the same time, indicates the limited capability of the low-resolution array of identifying the exact positions of small hotspots or coldspots. The junction tests as described in this work analyze the entire midlines of MLC junctions, but the method can be extended to analyses of individual leaf pair positions. The half-field junction tests with jaws were originally introduced to the linac QA in times of 3-D planning; nowadays, besides specials treatment plans where the beam-split technique is advantageous (e.g. for ovarian sparing of young women) they are important to define and check the absolute zero position in x and y direction that is relevant for other tests like the picket fence test where only relative positions (differences) are checked. With the 4-quadrant junction tests, on top of the leaf positioning, the dosimetric leaf gap stability can be checked. In MLC transmission tests, the array performance is limited compared to a high resolution 2-D detector, but it can still detect hotspots if the tolerance levels are adjusted. The array delivers good results in beam profile and beam energy stability tests. The suggestion of *OAR* or flatness as the beam energy metric is in accordance with Gao [8].

The procedures have been tested on various machines (different manufacturers, both C-arm and O-ring linacs). Modifications were necessary to reflect different collimator characteristics. Additional settings for junction tests were needed on the Novalis Tx to verify the backup jaw (X-jaw) position. Both Varian linacs do not allow for collimator rotation to 180° – here, 45° and 225° collimator angles have been combined to test the X-jaw (Novalis Tx only) and the MLC (Novalis Tx and Halcyon) sag at the gantry angle of 90°. The Vero does not support any collimator rotation – here, to test the MLC sag, gantry angles of 90° and 270° have been combined. The dual-layer MLC must be considered at Halcyon in further analyses, not considered in the current study.

In the other tests (MLC transmission, beam profile and beam energy constancy), the collimator properties do not affect the test selection. On the other hand, the variety of clinical beam energies must be considered. The MLC transmission must be tested with every clinical energy – at least at one gantry and collimator angle. In addition, it should be tested at other gantry and collimator angles with at least one clinical beam energy. The beam profile and beam energy constancy tests should be performed with each clinical beam energy to their full extent.

There are several other linac QA tests recommended by the AAPM TG-142 [4] or the German DIN 6847-5 standard [14] that can be performed with a 2-D array, but have not been analyzed in our work. Ritter [3], Skinner [5], and Kantz [6] have shown that a 2-D array can be deployed to test the output constancy or the output linearity. At our department, daily check devices and single ionization chambers are the tools of choice in these tests for practical reasons. In addition, the work of Ritter has shown the feasibility of MatriXX Evolution for the checks of various radiation source and collimating system parameters, the above-mentioned QA protocols [4], [14], [15] are not (or only partially) dealing with. These tests might be beneficial for identifying drifts in linac performance or changes after minor or major repairs [3].

## 5 Conclusions

We have replaced film dosimetry by 2-D array measurements in various types of linac QA tests. We have validated these tests and evaluated their sensitivity and specificity. Wherever reasonable, we have analyzed different metrics and suggested the most sensitive and specific ones. The results have shown that all tests can be implemented in the routine clinical QA program with considerable QA time savings.

## Author Contribution Statement

JV conceived the original idea. MS and CB helped design the experiments. JV carried out the measurements with support of MS. JV performed the calculations and data analyses. MS and CB contributed to the interpretation of the results. JV drafted the manuscript. MS and CB revised the manuscript critically. CB supervised the project. All authors approved the final version to be published and agree to be accountable for all aspects of the work in ensuring that questions related to the accuracy or integrity of any part of the work are appropriately investigated and resolved.

## Data Availability Statement

The supplementary material contains a list of suggested radiation fields. The code to process the data can be shared upon reasonable request. No patient data involved.

## Declaration of competing interest

The authors declare that they have no known competing financial interests or personal relationships that could have appeared to influence the work reported in this paper.
