# Supplementary material for: Implementation and validation of 2-D-array based tests in routine linac quality assurance
Source: Z Med Phys. 2024 Feb 16;35(3):270–81. doi: 10.1016/j.zemedi.2024.01.006 (PMC12664483; doi:10.1016/j.zemedi.2024.01.006)
Supplement: Supplementary Data 1 [file mmc1.pdf]

Implementation and validation of 2-D-array based tests in routine linac quality assurance: List of (suggested) irradiation fields for various linac types.

Supplementary material

TABLE S1. Set of irradiation fields for a multiple-energy single-layer-MLC linac with one pair of backup jaws and full collimator rotation (Versa HD).

Field description	Field size in cm²	Gantry angle	Collimator angle	Set of beam energies	Number of monitor units	Build-up	Used for
Reference open field	24.0 × 24.0	0°	0°	All clinically used	50	RW3 slabs	Junction & transmission tests
Bottom-left plus top-right		0°	0°	All clinically used	50	RW3 slabs	Junction (4 quadrants) tests
Bottom-right plus top-left		0°	0°	All clinically used	50	RW3 slabs	
Reference open field	24.0 × 24.0	90°	0°	6 MV	50	RW3 slabs	Junction (half field) tests
Y1-jaw		90°	90°	6 MV	50	RW3 slabs	
Y1-jaw		90°	270°	6 MV	50	RW3 slabs	
Y2-jaw		90°	270°	6 MV	50	RW3 slabs	
Y2-jaw		90°	90°	6 MV	50	RW3 slabs	
X1-leaves		90°	0°	6 MV	50	RW3 slabs	
X1-leaves		90°	180°	6 MV	50	RW3 slabs	
X2-leaves		90°	180°	6 MV	50	RW3 slabs	
X2-leaves		90°	0°	6 MV	50	RW3 slabs	
MLC overtravel left		0°	0°	All clinically used	50	RW3 slabs	MLC transmission tests
MLC overtravel right		0°	0°	All clinically used	50	RW3 slabs	
MLC interdigitation		0°	0°	All clinically used	50	RW3 slabs	
MLC overtravel left		90°	0°	6 MV	50	RW3 slabs	
MLC overtravel right		90°	0°	6 MV	50	RW3 slabs	
MLC interdigitation		90°	0°	6 MV	50	RW3 slabs	
MLC overtravel left		90°	90°	6 MV	50	RW3 slabs	
MLC overtravel right		90°	90°	6 MV	50	RW3 slabs	
MLC interdigitation		90°	90°	6 MV	50	RW3 slabs	
Open field	24.0 × 24.0	90°	0°	All clinically used	100	PEVP*	Beam profile tests
Open field	24.0 × 24.0	270°	0°	All clinically used	100	PEVP*	
Reference open field	24.0 × 24.0	0°	0°	All clinically used	100	PEVP*	Beam profile & energy tests
Open field	24.0 × 24.0	0°	0°	All clinically used	15	PEVP*	
Open field	24.0 × 24.0	0°	0°	All clinically used	5	PEVP*	

TABLE S2. Set of irradiation fields for a multiple-energy single-layer-MLC linac with two pairs of backup jaws and limited collimator rotation (Novalis Tx).

Field description	Field size in cm^2	Gantry angle	Collimator angle	Set of beam energies	Number of monitor units	Build-up	Used for
Reference open field	24.0 × 24.0	0°	0°	All clinically used	50	RW3 slabs	Junction & transmission tests
Bottom-left plus top-right		0°	0°	All clinically used	50	RW3 slabs	Junction (4 quadrants) tests
Bottom-right plus top-left		0°	0°	All clinically used	50	RW3 slabs	
Reference open field	24.0 × 24.0	90°	0°	6 MV	50	RW3 slabs	Junction (half field) tests
Reference open field	24.0 × 24.0	90°	45°	6 MV	50	RW3 slabs	
Y1-jaw		90°	90°	6 MV	50	RW3 slabs	
Y1-jaw		90°	270°	6 MV	50	RW3 slabs	
Y2-jaw		90°	270°	6 MV	50	RW3 slabs	
Y2-jaw		90°	90°	6 MV	50	RW3 slabs	
X1-jaw		90°	45°	6 MV	50	RW3 slabs	
X1-jaw		90°	225°	6 MV	50	RW3 slabs	
X2-jaw		90°	225°	6 MV	50	RW3 slabs	
X2-jaw		90°	45°	6 MV	50	RW3 slabs	
X1-leaves		90°	45°	6 MV	50	RW3 slabs	
X1-leaves		90°	225°	6 MV	50	RW3 slabs	
X2-leaves		90°	225°	6 MV	50	RW3 slabs	
X2-leaves		90°	45°	6 MV	50	RW3 slabs	
MLC overtravel left		0°	0°	All clinically used	50	RW3 slabs	MLC transmission tests
MLC overtravel right		0°	0°	All clinically used	50	RW3 slabs	
MLC interdigitation		0°	0°	All clinically used	50	RW3 slabs	
MLC overtravel left		90°	0°	6 MV	50	RW3 slabs	
MLC overtravel right		90°	0°	6 MV	50	RW3 slabs	
MLC interdigitation		90°	0°	6 MV	50	RW3 slabs	
MLC overtravel left		90°	90°	6 MV	50	RW3 slabs	
MLC overtravel right		90°	90°	6 MV	50	RW3 slabs	
MLC interdigitation		90°	90°	6 MV	50	RW3 slabs	
Open field	24.0 × 24.0	90°	0°	All clinically used	100	PEVP*	Beam profile tests
Open field	24.0 × 24.0	270°	0°	All clinically used	100	PEVP*	
Reference open field	24.0 × 24.0	0°	0°	All clinically used	100	PEVP*	Beam profile & energy tests
Open field	24.0 × 24.0	0°	0°	All clinically used	15	PEVP*	
Open field	24.0 × 24.0	0°	0°	All clinically used	5	PEVP*	

TABLE S3. Suggested set of irradiation fields for a single-energy dual-layer-MLC linac without backup jaws and limited collimator rotation (Halcyon).

Field description	Field size in cm²	Gantry angle	Collimator angle	Set of beam energies	Number of monitor units	Build-up	Used for
Reference open field	24.0 × 24.0	0°	0°	6 MV	50	RW3 slabs	Junction & transmission tests
Distal MLC<br>Bottom-left plus top-right		0°	0°	6 MV	50	RW3 slabs	Junction (4 quadrants) tests
Distal MLC<br>Bottom-right plus top-left		0°	0°	6 MV	50	RW3 slabs	
Proximal MLC<br>Bottom-left plus top-right		0°	0°	6 MV	50	RW3 slabs	
Proximal MLC<br>Bottom-right plus top-left		0°	0°	6 MV	50	RW3 slabs	
Reference open field	24.0 × 24.0	90°	0°	6 MV	50	RW3 slabs	Junction (half field) tests
Reference open field	24.0 × 24.0	90°	45°	6 MV	50	RW3 slabs	
Distal MLC X1-leaves		90°	45°	6 MV	50	RW3 slabs	
Distal MLC X1-leaves		90°	225°	6 MV	50	RW3 slabs	
Distal MLC X2-leaves		90°	225°	6 MV	50	RW3 slabs	
Distal MLC X2-leaves		90°	45°	6 MV	50	RW3 slabs	
Proximal MLC X1-leaves		90°	45°	6 MV	50	RW3 slabs	
Proximal MLC X1-leaves		90°	225°	6 MV	50	RW3 slabs	
Proximal MLC X2-leaves		90°	225°	6 MV	50	RW3 slabs	
Proximal MLC X2-leaves		90°	45°	6 MV	50	RW3 slabs	
Distal MLC overtravel left		0°	0°	6 MV	50	RW3 slabs	MLC transmission tests
Distal MLC overtravel right		0°	0°	6 MV	50	RW3 slabs	
Distal MLC interdigitation		0°	0°	6 MV	50	RW3 slabs	
Distal MLC overtravel left		90°	0°	6 MV	50	RW3 slabs	
Distal MLC overtravel right		90°	0°	6 MV	50	RW3 slabs	
Distal MLC interdigitation		90°	0°	6 MV	50	RW3 slabs	
Distal MLC overtravel left		90°	90°	6 MV	50	RW3 slabs	
Distal MLC overtravel right		90°	90°	6 MV	50	RW3 slabs	
Distal MLC interdigitation		90°	90°	6 MV	50	RW3 slabs	
Proximal MLC overtravel left		0°	0°	6 MV	50	RW3 slabs	
Proximal MLC overtravel right		0°	0°	6 MV	50	RW3 slabs	
Proximal MLC interdigitation		0°	0°	6 MV	50	RW3 slabs	
Proximal MLC overtravel left		90°	0°	6 MV	50	RW3 slabs	
Proximal MLC overtravel right		90°	0°	6 MV	50	RW3 slabs	
Proximal MLC interdigitation		90°	0°	6 MV	50	RW3 slabs	
Proximal MLC overtravel left		90°	90°	6 MV	50	RW3 slabs	
Proximal MLC overtravel right		90°	90°	6 MV	50	RW3 slabs	
Proximal MLC interdigitation		90°	90°	6 MV	50	RW3 slabs	
Open field	24.0 × 24.0	90°	0°	6 MV	100	PEVP*	Beam profile tests
Open field	24.0 × 24.0	270°	0°	6 MV	100	PEVP*	
Reference open field	24.0 × 24.0	0°	0°	6 MV	100	PEVP*	Beam profile & energy tests
Open field	24.0 × 24.0	0°	0°	6 MV	15	PEVP*	
Open field	24.0 × 24.0	0°	0°	6 MV	5	PEVP*	

TABLE S4. Set of irradiation fields for a single-energy single-layer-MLC linac without backup jaws and no collimator rotation (Vero).

Field description	Field size in cm²	Gantry angle	Collimator angle	Set of beam energies	Number of monitor units	Build-up	Used for
Reference open field	15.0 × 15.0	0°		6 MV	50	RW3 slabs	Junction & transmission tests
Bottom-left plus top-right		0°		6 MV	50	RW3 slabs	Junction (4 quadrants) tests
Bottom-right plus top-left		0°		6 MV	50	RW3 slabs	
Reference open field	15.0 × 15.0	90°		6 MV	50	RW3 slabs	Junction (half field) tests
Reference open field	15.0 × 15.0	270°		6 MV	50	RW3 slabs	
X1-leaves		90°		6 MV	50	RW3 slabs	
X1-leaves		270°		6 MV	50	RW3 slabs	
X2-leaves		270°		6 MV	50	RW3 slabs	
X2-leaves		90°		6 MV	50	RW3 slabs	
MLC overtravel left		0°		6 MV	50	RW3 slabs	MLC transmission tests
MLC overtravel right		0°		6 MV	50	RW3 slabs	
MLC interdigitation		0°		6 MV	50	RW3 slabs	
MLC overtravel left		90°		6 MV	50	RW3 slabs	
MLC overtravel right		90°		6 MV	50	RW3 slabs	
MLC interdigitation		90°		6 MV	50	RW3 slabs	
Open field	15.0 × 15.0	90°		6 MV	100	PEVP*	Beam profile tests
Open field	15.0 × 15.0	270°		6 MV	100	PEVP*	
Reference open field	15.0 × 15.0	0°		6 MV	100	PEVP*	Beam profile & energy tests
Open field	15.0 × 15.0	0°		6 MV	15	PEVP*	
Open field	15.0 × 15.0	0°		6 MV	5	PEVP*	

* PEVP – Photon Energy Verification Plate.
